# CoreNEURON : An Optimized Compute Engine for the NEURON Simulator

**DOI:** 10.3389/fninf.2019.00063

**Published:** 2019-09-19

**Authors:** Pramod Kumbhar, Michael Hines, Jeremy Fouriaux, Aleksandr Ovcharenko, James King, Fabien Delalondre, Felix Schürmann

**Affiliations:** ^1^Blue Brain Project, École Polytechnique Fédérale de Lausanne (EPFL), Geneva, Switzerland; ^2^Department of Neuroscience, Yale University, New Haven, CT, United States

**Keywords:** NEURON, simulation, neuronal networks, supercomputing, performance optimization

## Abstract

The NEURON simulator has been developed over the past three decades and is widely used by neuroscientists to model the electrical activity of neuronal networks. Large network simulation projects using NEURON have supercomputer allocations that individually measure in the millions of core hours. Supercomputer centers are transitioning to next generation architectures and the work accomplished per core hour for these simulations could be improved by an order of magnitude if NEURON was able to better utilize those new hardware capabilities. In order to adapt NEURON to evolving computer architectures, the compute engine of the NEURON simulator has been extracted and has been optimized as a library called CoreNEURON. This paper presents the design, implementation, and optimizations of CoreNEURON. We describe how CoreNEURON can be used as a library with NEURON and then compare performance of different network models on multiple architectures including IBM BlueGene/Q, Intel Skylake, Intel MIC and NVIDIA GPU. We show how CoreNEURON can simulate existing NEURON network models with 4–7x less memory usage and 2–7x less execution time while maintaining binary result compatibility with NEURON.

## 1. Introduction

Simulation in modern neuroscientific research has become a third pillar of the scientific method, complementing the traditional pillars of experimentation and theory. Studying models of brain components, brain tissue or even whole brains provides new ways to integrate anatomical and physiological data and allow insights into causal mechanisms crossing scales and linking structure to function. Early studies covered for example the levels from channels to cell behavior accounting for detailed morphology (e.g., De Schutter and Bower, 1994; Mainen and Sejnowski, 1996) and integrating this detail into models of networks (e.g., Davies, 1992). More recently, studies have been accounting for increased electrophysiological detail and diversity in the tissue model (e.g., Markram et al., 2015; Arkhipov et al., 2018), giving a glimpse at functional importance of the underlying connectome (e.g., Gal et al., 2017; Reimann et al., 2017) allowing for example the reinterpretation of aggregate brain signals such as LFP (e.g., Anastassiou et al., 2015). At the same time, computational studies have strived to look even deeper into the biochemical workings of the cell, studying the role of intracellular cascades in neuromodulation (e.g., Lindroos et al., 2018) or metabolism (e.g., Jolivet et al., 2015), and to abstract some of the detail while maintaining cell type diversity (e.g., Izhikevich and Edelman, 2008; Potjans and Diesmann, 2012; Dahmen et al., 2016), or to move the integrated and modeled data all the way to fMRI (Deco et al., 2008).

As the biochemical and biophysical processes of the brain span many orders of magnitudes in space and time, different simulator engines have been established over time incorporating the appropriate idioms, computational representations and numerical methods (e.g., at the biochemical level—STEPS Wils and De Schutter, 2009, at the detailed cellular level - NEURON Migliore et al., 2006, using simplified neuron representations—NEST Gewaltig and Diesmann, 2007, or even more abstract—TVB Sanz-Leon et al., 2015 to name a few).

The more detail is included in these models and the larger the models become, the larger are the computational requirements of these simulation engines, making it necessary to embrace advanced computational concepts and faster computers (Hines et al., 2011; Hepburn et al., 2016; Ippen et al., 2017). Table 1 shows exemplarily five different network models used in this paper for benchmarking and indicates their size and complexity.

**Table 1:** Summary of network models.

**Model name**	**Summary**	**#Neurons**	**#Compartments**	**#Synapses**
Traub	A single column thalamocortical network model	3,560	465,740	1,099,820
Dentate	Dentate Gyrus model including Granule cells with dendritic compartments	5,137	175,719	1,199,988
Ring	Ring network of branching cells	32,768	9,535,488	33,280
Cortex + Plasticity	Somatosensory cortex model with synaptic plasticity	219,422	99,581,138	872,922,040
Hippocampus	Rat Hippocampus CA1 model	789,595	565,495,731	361,937,388

A single-column thalamocortical network model (Traub et al., 2005) is used to better understand population phenomena in thalamocortical neuronal ensembles. It has 3,560 multi-compartment neurons with soma, branching dendrites and a portion of axon. It consists of 14 different neuron types, 3,500 gap junctions and 1.1 million connections. The neurons were connected together by chemical synapses (using AMPA and NMDA receptors) and gap junctions that were non-rectifying and voltage-independent. This model uses standard repertoire of 11 active conductances in all of the cells. A scaled-down variant of the full-scale dentate gyrus model (Dyhrfjeld-Johnsen et al., 2007) developed in the (Soltesz Lab, 2019) is used to understand hippocampal spatial information processing and field potential oscillations. It consists of 5,143 multi-compartment neurons and 4,121 Poisson spike sources, and includes 6 different cell types, 1.2 million connections and about 600 gap junctions. This model uses 9 classes of active conductance mechanisms such as sodium, potassium, calcium channels, and calcium-dependent potassium channels. A synthetic model with specific computational characteristics is often needed to evaluate target hardware based on number of cells, branching patterns, compartments per branch etc. For this purpose, a multiple ring network model of branching neurons and minimal spike overhead is used (Hines, 2017a). The Blue Brain Project has published a first-draft digital reconstruction of the microcircuitry of somatosensory cortex in 2015 (Markram et al., 2015). This model contains about 219,000 neurons, with 55 layer-specific morphological and 207 morpho-electrical neuron subtypes. The neurons in this model employ up to 13 different Hodgkin-Huxley conductance classes, with up to 8 of those classes used in the dendrites. Together with other partners in the European Human Brain Project, this group is also working on a full-scale model of a rat hippocampus CA1 (Human Brain project, 2018). A first draft of this model contains about 789,000 neurons with 13 morphological types and 17 morpho-electrical types. The neurons in this model employ up to 11 active conductance classes, with up to 9 of those classes used in the dendrites.

The number of neurons and synapses, however, is not always the best indicator of the computational complexity of a model. In the model of Markram et al. (2015) each neuron averages to about 20,000 differential equations to represent its electrophysiology and connectivity. To simulate the microcircuit of 31,000 neurons, it is necessary to solve over 600 million equations every 25 ms of biological time–a requirement far beyond the capabilities of any standard workstation. It is necessary to utilize massively parallel systems for such simulations but fully exploiting the capabilities these systems is a challenging task for a large number of scientific codes, including NEURON. Significant efforts are necessary to prepare scientific applications to fully exploit the massive amount of parallelism and hardware capabilities offered by these new systems (Ábrahám et al., 2015).

In this paper we present our efforts to re-engineer the internal computational engine of the NEURON simulator, CoreNEURON, to adapt to emerging architectures while maintaining compatibility with existing NEURON models developed by the neuroscience community. Our work was guided by the goal to leverage the largest available supercomputers for neuroscientific exploration by scaling the simulator engine to run on millions of threads. A key design goal was to reduce the memory footprint compared to NEURON as total memory and memory bandwidth are scarce and costly resources when running at scale. Lastly, for this capability to be easily usable by the normal NEURON community, we endeavored to tightly integrate CoreNEURON with NEURON.

## 2. NEURON Simulation Environment

NEURON is a simulation environment developed over the last 35 years for modeling networks of neurons with complex branched anatomy and biophysical membrane properties. This includes extracellular potential near membranes, multiple channel types, inhomogeneous channel distribution and ionic accumulation. It can handle diffusion-reaction models and integrating diffusion functions into models of synapses and cellular networks. Morphologically detailed models simulated using NEURON are able to represent the spatial diversity of electrical and biophysical properties of neurons.

Individual neurons are treated as a tree of unbranched cables called *sections*. Each section can have its own set of biophysical parameters, independently from other sections, and is discretized as a set of adjacent *compartments* (see e.g., Hines, 1993). Compartmental models of neurons take into account not only the connectivity between neurons but also the individual morphologies and inhomogeneities of each neuron. The electrical activity of neurons is modeled using the cable equation (see e.g., Tuckwell, 2005) applied to each section, where the quantity representing the state of a neuron at a given point in space and instant in time is the *membrane potential*. The general form of the cable equation for a section, in the case of constant parameters and conductance based synapse modeling, is given by:

(1) $$\begin{array}{l} \frac{d}{4R_{a}}\frac{\partial^{2}v}{\partial x^{2}}=c_{m}\frac{\partial v}{\partial t}+I_{pas}+I_{ion}+I_{syn} \end{array}$$

where

- $d\left[\mu m\right],R_{a}\left[\Omega cm\right],c_{m}\left[\frac{\mu F}{cm^{2}}\right],I_{pas}\left[\frac{mA}{cm^{2}}\right]$ are biophysical parameters contributing to the passive component of the cable equation (unit conversion factors are not shown but each term has the units of *mA*/*cm*^2^).
- $I_{ion}\left[\frac{mA}{cm^{2}}\right]$ is the active contribution arising from ion channels along the section, whose conductances *g*_*i*_ and resting potentials *e*_*i*_ might depend in a non-linear fashion upon a set of state variables representing those channels.
- $I_{syn}\left[\frac{mA}{cm^{2}}\right]$ is the contribution from the synapses placed at positions *x*_*j*_, whose conductances *g*_*j*_ and resting potentials *e*_*j*_ might depend in a non-linear fashion upon a set of state variables and which take effect in a strongly localized manner. Individual synapses have units of *nA* and conversion to *mA*/*cm*^2^ involves a Dirac delta function, δ(*x*−*x*_*j*_), with units 1/μ*m*, and the diameter; i.e., conversion of absolute current to current per unit area implies division by the compartment area where the synapse is located.

One needs to couple (1) to a set of additional differential equations that describe the evolution of the states of ion channels and synapses, thus giving rise to a system of PDEs/ODEs as the final problem. Spatial discretization of the PDEs results in a tree topology set of stiff coupled equations which is most effectively solved by implicit integration methods. In particular, direct Gaussian elimination with minimum degree ordering is computationally optimum in the sense that the number of arithmetic operations is identical to direct Gaussian elimination of a non-branching cable with the same number of nodes (Hines and Carnevale, 1997; Hines et al., 2008). The general structure of a *hybrid clock-event driven algorithm* (Hines, 1993) in NEURON can be divided into a set of operations that are performed at every integration time step and an interprocess spike exchange operation where a list of spike generation times and identifiers are synchronized across all processors every minimum spike delay interval. The per integration step operations are:

- Event-driven spike delivery step where the callback function of each synapse activated by a spike at a given timestep is executed.
- Matrix assembly step where the *I*_*ion*_ and *I*_*syn*_ contributions are computed and included in the matrix.
- Matrix resolution step where the membrane potential for the current step is obtained by solving a linear system.
- State variables update step where the evolution equations for the states of ion channels and synapses are solved to advance to the current timestep.
- Threshold detection step where each neuron is scanned to see if it has met a particular firing condition, and if so a particular list of events is updated.

Although the simulator has demonstrated scaling up to 64,000 cores on the IBM Blue Gene/P system (Hines et al., 2011), with the emerging computing architectures (like GPUs, many-core architectures) the key challenges are numerical efficiency and scalability. The simulator needs to : (1) expose fine grain parallelism to utilize the massive number of hardware cores, (2) be optimized for memory hierarchies and (3) fully utilize processor capabilities such as vector units. To simulate models with billions of neurons on a given computing resource, memory capacity is another major challenge. In order to address these challenges, the compute algorithm of the NEURON simulator was extracted and optimized into a standalone library called CoreNEURON.

## 3. CoreNEURON Design and Implementation

The integration interval operations (listed in section 2) consume most of the simulation time (Kumbhar et al., 2016). The goal of CoreNEURON is to efficiently implement these operations considering different hardware architectures. This section describes the integration of CoreNEURON with the NEURON execution workflows, major data structure changes to reduce memory footprint, memory transfer between NEURON-CoreNEURON and a checkpoint-restore implementation to facilitate long running simulations.

### 3.1. NEURON to CoreNEURON Workflow

**Figure 1:**
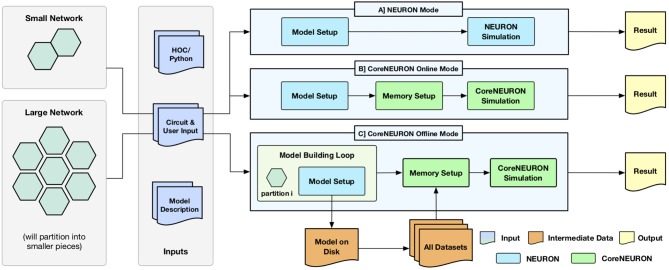
Different execution workflows supported by NEURON simulator with CoreNEURON : (A) shows the existing simulation workflow where HOC/Python interface is used for building a model which is then simulated by NEURON; (B) shows the new CoreNEURON based workflow where the in-memory model constructed by NEURON is transferred using direct memory access and then simulated by CoreNEURON; (C) shows new CoreNEURON based workflow where NEURON partitions a large network model into smaller chunks, iteratively instantiates each model piece in memory, and copies that subset of model information to disk. CoreNEURON then loads the whole model in memory and simulates it.

One of the key design goal of CoreNEURON is to be compatible with the existing NEURON models and user workflows. With the integration of CoreNEURON library, the NEURON simulator supports three different workflows depicted in Figure 1.

- NEURON mode
- CoreNEURON Online mode
- CoreNEURON Offline mode

Existing users are familiar with the default *NEURON mode*. The model descriptions written in NMODL (Hines and Carnevale, 2000) are used to build a dynamically loadable shared library. The HOC/Python scripting interface is used to build a network model in memory (*Model Setup phase*). This in-memory model is then simulated using the *hybrid clock-event driven algorithm* described in section 2 (*Simulation phase*). Users have full control over model structure and can introspect or record all events, states, and model parameters using the scripting or graphical user interface (*Result phase*).

*CoreNEURON Online Mode* allows users to run their models efficiently with minimal changes. After the *Model Setup phase*, the in-memory representation is copied into CoreNEURON's memory space. CoreNEURON then re-organizes the memory during *Memory Setup phase* for efficient execution (see section 4.2). The *Simulation phase* is executed in CoreNEURON and spike results are written to disk. Note that the same NMODL model descriptions are used both in NEURON as well as CoreNEURON.

*CoreNEURON Offline* mode is intended for large network models that cannot be simulated with NEURON due to memory capacity constraints. In this mode, instead of loading the entire model at once, the *Model Setup phase* builds a subset of the model that fits into available memory. That subset is written to disk, the memory used by the subset is freed, and the *Model Setup phase* constructs another subset. After all subsets are written by NEURON, CoreNEURON reads the entire model from the disk and begins the *Simulation phase*. Because CoreNEURON's cell and network connection representations are much lighter weight than NEURON's, 4-7x larger models than NEURON can be simulated with CoreNEURON (see section 5).

Users can adapt existing models to the *CoreNEURON Online Mode* workflow with the trivial replacement of the *psolve* function call with *nrncore_run* of the (ParallelContext, 2019) class.

### 3.2. Data Structure Changes

NEURON is used as a general framework for designing and experimenting with neural models of varying anatomical detail and membrane complexity. Users can interactively create cells with branches of varying diameters and lengths, insert ionic channels, create synapses, and visualize different properties using a GUI. In order to provide this introspection capability, NEURON maintains a large number of complex data structures. Typically, once the users are satisfied with the behavior of the model, they run larger/longer simulations on workstations or clusters where those interactivity or detailed introspection capabilities are often no longer required. In this type of batch execution, memory overhead from many large, complex data structures with many mutual pointers can be significantly reduced by replacing them with fixed arrays of data structures in which the few necessary pointers are replaced by integers. For example, the network connection object (*Netcon*) and the common synapse base class (*Point_process*), which are responsible for a significant portion of memory usage in NEURON, were reduced from 56 to 40 and 56 to 8 bytes respectively in CoreNEURON. Table 2 lists the important data structures and their memory usage comparison between NEURON and CoreNEURON. CoreNEURON eliminates the Python/HOC interpreter and so, datastructures like *Node, Section, Object* are no longer needed. The memory usagemprovements from these optimizations for different network models are discussed in section 5.

**Table 2:** Memory footprint comparison for different data structures (in bytes).

**Data structure**	**Purpose**	**NEURON**	**CoreNEURON**
Node	Compartment of the neuron	128	–
Section	Unbranched cable of the neuron	96	–
Object	High level HOC object	64	–
Presyn	Synapse object at origin	208	64
InputPresyn	Similar to Presyn	–	24
Point_process	Synapse overhead	56	8
Prop	Property object in compartment	48	–
Netcon	Connection between neuron	56	40
Pointer	Memory address	8	4
Memb_list	List of mechanisms or channels	56	64
NrnThreadMembList	Mechanism list for group of neurons	34	40
PreSynHelper	Helper object for PreSyn	–	4
Symbol	Token parsed by HOC interpreter	56	–

### 3.3. Pointer Semantics

NEURON users can define their own data structures and allocate memory through the use of *POINTER* and *VERBATIM* constructs of NMODL (Hines, 2019). Many internal data structures of NEURON use pointer variables to manage various dynamic properties, connections, event queues etc. As a model is built incrementally using the scripting interface, various memory pools are allocated during the *Model Setup phase*. As data structures between NEURON and CoreNEURON are different, serializing memory pools becomes one of the major memory management challenges of the CoreNEURON implementation. With serialization, pointer variables need to be augmented with meta information to allow proper decoding by CoreNEURON. This meta information indicates the *pointer semantics*. All data variables which potentially are the target pointers are grouped into a contiguous memory pool and pointer variables are converted to an integer offset into the memory pool. When the NEURON pointers are copied to CoreNEURON's memory space, the semantic type associated with the pointer variable is used to compute the corresponding integer offset. Different semantic types with their purpose are listed in Supplementary Material (see Table S1).

### 3.4. Checkpoint-Restart Support

**Figure 2:**
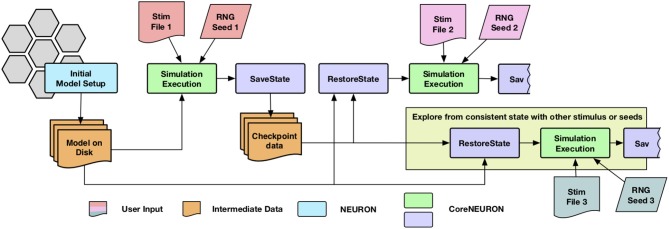
Simulation workflow with the checkpoint-restart feature : CoreNEURON loads the model from disk, simulate it and dumps in-memory state back to disk (*SaveState* step). CoreNEURON can load checkpoint data (*RestoreState* step) and continue the simulation on a different machine using the checkpoint data. The user has flexibility to launch multiple simulations with different stimuli or random number streams (*Stim* or *RNG*) in order to explore network stability and robustness.

The network simulations for studying synaptic plasticity can run from a week to a month. Enabling such simulations of long biological time-scales is one of the important use cases for CoreNEURON. Most of the cluster and supercomputing resources have a maximum wall clock time limit for a single job (e.g., up to 24 h). The checkpoint-restart (Schulz et al., 2004) is commonly used technique to enable long running simulations and has been implemented in CoreNEURON. Since the checkpoint operation could take place at anytime with varying degrees of cell firing activity, it was necessary to account for generated yet undelivered synaptic events in addition to saving the in-memory state of the simulator. When a cell fires, it may have many connections to other cells with different delivery delays. During the checkpoint operation, any undelivered messages are collapsed back into the original event of the firing cell so that a single event can be saved. Once the network simulation is checkpointed, users have flexibility to launch multiple simulations with different stimuli or random number streams in order to explore network stability and robustness. The execution workflow of such simulations is shown in Figure 2.

### 3.5. Spike Communication

In CoreNEURON, the MPI communication and event queue handling for spike delivery is inherited from NEURON and remains on the CPU. Performance of those components is discussed in Kumar et al. (2010); Hines et al. (2011). However, when GPUs are in use, all the spikes within a time step that are destined for a specific synapse type are copied to the GPU to a type specific buffer and thereafter all NET_RECEIVE block computations take place on the GPU. Conversely, threshold detection takes place on the GPU as well and spike generation is buffered until the end of the time step at which point the buffer spikes are copied to the CPU for MPI transfer and enqueueing onto the priority queue. The exception to this strategy is that ARTIFICIAL_CELL instances, which compute and generate spikes solely by their NET_RECEIVE block response to delivered events, exist only on the CPU.

### 3.6. Portability Considerations

CoreNEURON can transparently handle all spiking network simulations including gap junction coupling with the fixed time step method. The model descriptions written in NMODL need to be (THREADSAFE, 2019) to exploit vector units of modern CPUs and GPUs. A model can be non thread-safe if a MOD file contains GLOBAL variables which are used for temporary storage by getting assigned a value in one procedure and evaluated in another. Such variables need to be converted from GLOBAL to RANGE. This can be achieved with the help of NEURON's *mkthreadsafe* tool or the user can manually make the minor change to such MOD files. New keywords like *COREPOINTER* and *CONDUCTANCE* have been added to NMODL to facilitate serialization and improve performance optimization respectively. These keywords are also backported to NEURON so that the models remain compatible for either NEURON or CoreNEURON execution. For scalability and portability of random numbers on platforms like GPUs, CoreNEURON supports the Random123 pseudo-random generator (Salmon et al., 2011).

## 4. Optimizations

In order to improve the performance of CoreNEURON on different architectures, different optimization schemes are implemented for multi-threading, memory layout, vectorization, and code generation. These optimizations are described in this section.

### 4.1. Parallelism

Both NEURON and CoreNEURON use the *Message Passing Interface* (MPI) to implement distributed memory parallelism. Although NEURON supports multi-threading based on Pthread (Nichols et al., 1996), users commonly use pure MPI execution due to better scaling behavior. But, pure MPI execution will affect scalability due to MPI communication and memory overhead of internal MPI buffers when executing at scale (Lange et al., 2013). To address this scalability and parallelism challenge, CoreNEURON relies on three distinct levels of parallelism. First, at the highest level, a set of neurons that have equivalent computational cost are grouped together and assigned to each MPI rank on the compute node. Second, within a node, an individual neuron group is assigned to an OpenMP (Dagum and Menon, 1998) thread executing on a core. This thread simulates the given neuron group for the entire simulation ensuring data locality. Finally, vector units of the core are utilized for executing groups of channels in parallel. With respect to MPI and OpenMP, simulations may benefit from fewer MPI processes per compute node (down to a single process per node). Based on target architecture, users can choose a number of MPI ranks and corresponding OpenMP threads per rank to reduce communication overhead.

### 4.2. Memory Layout and Vectorization

**Figure 3:**
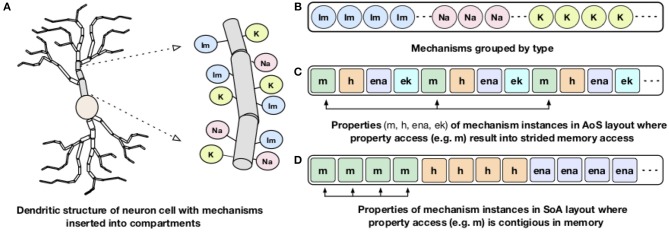
Dendritic structure and memory layout representation of a neuron: A schematic representation of dendritic structure of a neuron with different mechanisms inserted into the compartment is shown on the left **(A)**. On the right: **(B)** shows how NEURON and CoreNEURON groups the mechanism instances of the same type; **(C)** shows how NEURON stores properties of individual mechanism in the *AoS* layout; **(D)** shows the new *SoA* layout in CoreNEURON for storing mechanism properties.

Processor memory bandwidth is one of the scarce resources and often the major impediment to improve the performance of many applications including NEURON. The compute kernels of channels and synapses are bandwidth limited and can reach close-to-peak memory bandwidth (Kumbhar et al., 2016). The dendritic structures of a neuron are divided into small compartments and different membrane channels or mechanisms are inserted into different compartments (Figure 3A). For memory locality, both NEURON and CoreNEURON groups the channels by their type as shown in Figure 3B. But, NEURON organizes properties of individual mechanisms (like *m, h, ena*) in the *Array of Structs (AoS)* memory layout (Figure 3C). When a specific property is accessed, for example, *m*, it results in strided memory accesses with inefficient memory bandwidth utilization and hence poor performance. To address this issue, CoreNEURON organizes channel properties into the *Structure of Arrays (SoA)* memory layout (Figure 3D). This allows efficient vectorization and efficient memory bandwidth utilization for all channel and synapse computations. For code vectorization, CoreNEURON is dependent on the compiler's auto-vectorization capabilities. To assist the compiler in auto-vectorization, hints like *#pragma ivdep* are used. The performance improvements from this optimization is discussed in Kumbhar et al. (2016).

### 4.3. NMODL Source-to-Source Translator

**Figure 4:**
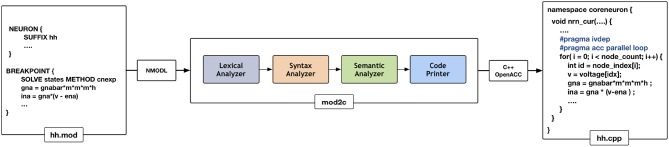
Code generation workflow for CoreNEURON : different phases of the source-to-source compiler are shown in the middle that translates the input model description file (hh.mod) to C++ code (hh.cpp). Compiler hints like *ivdep* and *acc parallel loop* are inserted to enable CPU vectorization/GPU parallelization.

NEURON has had support for code generation through the model description language, NMODL, since version 2 released in 1989 (Blundell et al., 2018). The code generation program of NEURON has been modified into a standalone tool called *MOD2C* (MOD2C GitHub Repository, 2019). This tool is used by CoreNEURON to support all NEURON models written in NMODL. Figure 4 shows the high level workflow of MOD2C. The first step of source-to-source translator is *lexical analysis* where lexical patterns in the NMODL code are detected and tokens are generated. The *syntax analysis* step uses those tokens and determine if the series of tokens are appropriate in the language. The *semantic analysis* step make sure if syntactically valid sentences are meaningful as part of the model description. *Code generation* is the step in which a C++ file is created with compiler hints for auto-vectorization (e.g., *#pragma ivdep*) and GPU parallelization with the OpenACC programming model (Wikipedia, 2012). MOD2C also takes care of code generation for *AoS* and *SoA* memory layouts. MOD2C uses open source flex and bison tools (Levine and John, 2009) for this implementation. More information about the NMODL code generation pipeline can be found in Blundell et al. (2018).

### 4.4. GPU Porting

**Figure 5:**
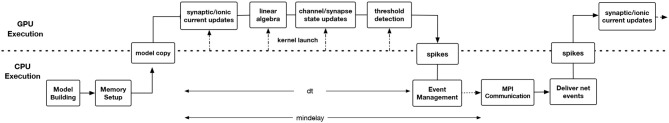
Timeline showing the workflow of GPU-enabled CoreNEURON execution. The *Model Building* and *Memory Setup* phases are executed on CPU by NEURON and CoreNEURON respectively. The latter performs an in-place memory AoS to SoA transformation and node permutation to optimize Gaussian elimination. The CoreNEURON in-memory model is then copied to GPU memory using *OpenACC* APIs. All time step integration phases including threshold detection for event generation and event delivery to synapse models take place on the GPU. At the end of each timestep (*dt*), the generated spike events are transferred to the CPU. Conversely, all the spike events to be delivered during a step are placed in a per-synapse type buffer and transferred at the beginning of each timestep to the GPU. At the end of *mindelay* interval all spikes destined to other processes are transferred using MPI Communication.

Prior to the CoreNEURON project, a substantial effort was made to port NEURON to the GPU architecture using the CUDA programming model (Wikipedia, 2006; NVIDIA Corporation, 2006–2017). One of the two major components of this implementation was the extension of the NMODL source-to-source compiler to emit CUDA code. The other major component managed an internal memory transformation from NEURON's thread efficient *AoS* memory layout to a more GPU memory efficient *SoA* layout. For generating CUDA code, there was a separate version of the NMODL source-to-source compiler. NEURON maintains complex data structures of section, segment for interactive use. The memory management of these *non-POD type* (Plain Old Data) data structures between CPU and GPU was quite complex as memory allocations were not contiguous. This experimental NEURON version (Hines, 2014) was quite efficient for matrix setup and channel state integration for cellular simulations but did not reach network simulation capability. The project foundered on software administration difficulties of maintaining two completely separate codebases, the difficulty of understanding the data structure changes involved for memory layout transformation from *AoS* to *SoA*, and the difficulty of managing pointer updates in the absence of pointer semantics information. It became clear that a more general view was required that could not only alleviate these problems for the GPU but had a chance of evolving to work on future architectures. This view is embodied in CoreNEURON development. As discussed in section 4.2, CoreNEURON data structures and memory layout have been optimized for efficient memory access. MOD2C supports code generation with the OpenACC programming model that helps to target different accelerator platforms. Users need to compile the CoreNEURON library with a compiler that supports OpenACC. Figure 5 shows GPU enabled execution workflow where different stages of the CoreNEURON simulator running on CPU and GPU are described.

**Figure 6:**
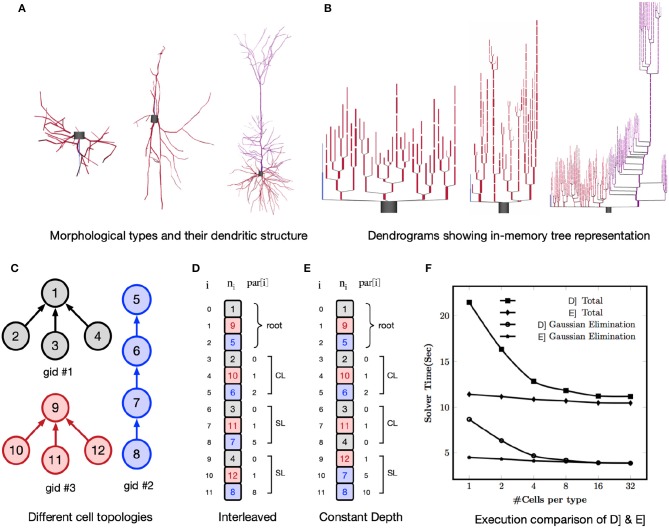
The top row shows three different morphological types with their dendritic tree structure in **(A)** and dendrograms showing in-memory tree representation of these types in CoreNEURON in **(B)**. The bottom row shows different node ordering schemes to improve the memory access locality on GPUs : **(C)** Example topologies of three cells with the same number of compartments; **(D)** Interleaved Layout where a compartment from each of N cells forms an adjacent group of N compartments. For *i*th node, *n*_i_ is node index and *par[i]* is its parent index. With three executor threads, square brace highlight parent indices that result into contiguous memory load (CL) and strided memory load (SL); **(E)** Constant Depth Layout where all nodes at same depth from root are adjacent; **(F)** Comparison of two node ordering schemes for Ring network model showing execution time of whole simulation and Gaussian Elimination step.

One of the performance challenges for a GPU implementation is irregular memory accesses due to the non-homogeneous tree structure of neurons. For example, Figure 6A shows three different morphological types and their compartmental tree connection topology in the simulator is shown in Figure 6B. The GPU delivers better performance when consecutive threads (in groups of 16 or 32) perform the same computations and load the data from consecutive memory addresses. When there are a large number of cells per morphological type, it is straightforward to achieve optimal performance by interleaving the compartments of identical cells. But, with few cells per morphological type, Gaussian elimination suffers from non-contiguous layout of parents relative to a group of nodes. This results in irregular, strided memory accesses and hence poor performance (Valero-Lara et al., 2017). To address this, two alternative node orderings schemes, *Interleaved* layout and *Constant Depth* layout, are implemented as illustrated in Figures 6D,E. All cells have the same number of compartments but each has a different branching pattern (Figure 6C). Nodes (representing compartments) within a cell are numbered with successive integers. In the case of *Interleaved* layout, a compartment from each of *N* cells forms an adjacent group of *N* compartments. The groups are in any root to leaf order but corresponding compartments in identical cells are adjacent. As an example, for a group of three threads the vertical square braces highlight parent indices that have the same order as the nodes. This results in either contiguous memory loads (*CL*) or strided memory load (*SL*). For each Gaussian elimination operation the number of threads that can compute in parallel is equal to the number of cells and hence this scheme is referred as *one cell per thread layout*. For *Constant Depth* layout, all nodes at the same depth from the root are adjacent. For a given depth, corresponding nodes of identical cells are adjacent. Children of branch nodes in the same cell are kept as far apart as possible to minimize contention while updating the same node from different threads.

To analyse the impact of node ordering schemes on the execution time, we used a multiple Ring network model of cells with random tree topology (Hines, 2017a). This test allows to evaluate performance impact when parents of a contiguous group of 32 nodes are not contiguous and executed in chunks of 32 threads (a so-called *warp*). We used a multiple Ring model with a total of 131,072 cells comprising 10,878,976 nodes running for 10 ms on NVIDIA K20X GPU (NVIDIA Corporation, 2012). Every cell has the same number (83) of nodes but different cell types have a different random branching pattern of the 40 dendrites. The number of identical cells per type ranges from 1 (131,072 distinct branching patterns) to 32 (4096 distinct branching patterns). Note that regardless of the branching pattern, Gaussian elimination takes exactly the same number of arithmetic operations. Figure 6D shows performance of Interleaved Layout and Constant Depth Layout. For both node ordering schemes, performance is optimal with regard to parent ordering when there are at least 32 cells of each type corresponding to the 32 threads operating in *Single Instruction Multiple Data (SIMD)* mode. With fewer cells per type, parent node ordering becomes less than optimal and the performance of Interleaved layout suffers by up to a factor of two. Note that the total runtime deteriorates more rapidly than Gaussian elimination time due to the fact that the parent contiguity also affects the performance of tree matrix setup during evaluation of a node's current balance equation. The execution time of *Constant Depth* layout shows that it is possible to permute node ordering so that parent nodes are more likely to be in significant conti guous order relative to their children. The constant ratio between total runtime and Gaussian elimination is due to negligible time contribution of passive dendrites to matrix setup in combination with the significant role of parent ordering in computing the effect of topologically adjacent nodes on matrix setup of the current balance equations.

## 5. Benchmarks and Performance

Not all network models are compute intensive or benefit equally from CoreNEURON optimizations. In order to evaluate the performance improvements with the optimizations discussed in the previous section we ran several published network models listed in Table 1 on different computing architectures. This section describes the benchmarking platforms and compares performance between NEURON and CoreNEURON.

The benchmarking systems with hardware details, compiler toolchains and network fabrics are summarized in Table 3. The Blue Brain IV (BB4) and Blue Brain V (BB5) systems are based on IBM BlueGene/Q (Haring et al., 2012) and HPE SGI 8600 (Hewlett Packard Enterprise, 2019) platforms respectively, hosted at the Swiss National Computing Center (CSCS) in Lugano, Switzerland. The BB4 system has 4,096 nodes comprising 65,536 PowerPC A2 cores. The BB5 system has three different compute nodes: Intel KNLs with low clock rate but high bandwidth MCDRAM, Intel Skylakes with high clock rate, and NVIDIA Volta GPUs. Vendor provided compilers and MPI libraries are used on both systems. The BB4 system is used for strong scaling benchmarks (see Figure 8) as it has a large core count compared to the BB5 system. All benchmarks were executed in pure MPI mode by pinning one MPI rank per core. During the model building phase, NEURON divides model into *n* equal chunks where *n* is total number of MPI ranks. CoreNEURON continues execution with the same number of MPI ranks as NEURON. For GPU executions we used one MPI rank per GPU node.

**Table 3:** Details of benchmarking systems.

BlueGene/Q (BB4)	Processor	IBM PowerPC A2, 16 cores @ 1.6 GHz, 16 GB DRAM
	Compiler toolchain	IBM XL 12.1 and IBM MPI
	Network	Integrated 5-D torus
Intel Skylake (BB5)	Processor	2 Xeon 6140, 36 cores @ 2.3 GHz, 384 GB DRAM
	Compiler toolchain	Intel 2018.1 and HPE-MPI (MPT)
	Network	InfiniBand EDR
Intel KNL (BB5)	Processor	Xeon Phi (7230), 64 cores @ 1.3 GHz, 96 GB DRAM
	Compiler toolchain	Intel 2018.1 and HPE-MPI (MPT)
	Network,	InfiniBand EDR
NVIDIA GPU (BB5)	Processor	NVIDIA GPU V100 SXM2, 2 Xeon 6140, 36 cores @ 2.3 GHz
	Compiler toolchain	PGI 18.10, OpenMPI 2.0
	Network	InfiniBand EDR

**Figure 7:**
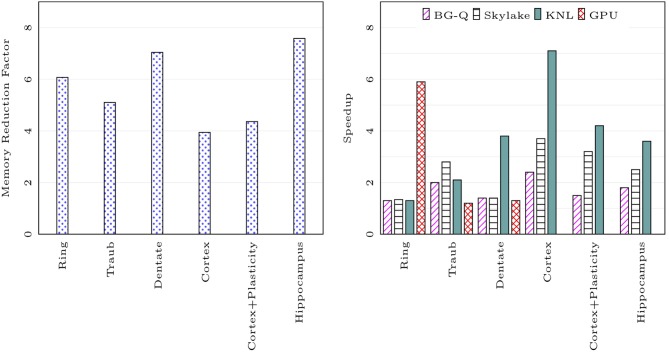
Memory usage reduction and speedup using CoreNEURON : ratios of memory usage between NEURON and CoreNEURON for different models in Table 1 are shown on the left (measured on BB4 system). Speedups of CoreNEURON simulations compared to NEURON on various architectures (using single node) for the same models are shown on the right.

We compared the memory footprint of different network models listed in Table 1. Figure 7 on the left shows memory usage reduction with CoreNEURON simulation compared to NEURON simulation. The memory reduction factor depends on various model properties (e.g., number of compartments, sections, synapses, etc.) but one can expect 4-7x reduction with the use of CoreNEURON. Note that *CoreNEURON Online mode* will need $\frac{1}{7}$x to $\frac{1}{4}$x more memory during the *Memory Setup phase*. But once the model is transferred to CoreNEURON for simulation, NEURON can free allocated memory.

Figure 7 on the right shows the speedup achieved on a single node for different models with CoreNEURON compared to NEURON. Note that the Cortex and Hippocampus models are very large in terms of memory capacity requirement. For single node performance analysis we used a smaller subset of these two models.

The memory layout and code vectorization optimization described in section 4.2 shows greatest improvement when most of the computation time is spent in channel and synapse computations. The Cortex, Cortex+Plasticity and Hippocampus models have cells with 200 to 800 compartments and 20 different channel types. This makes these models compute intensive and lets them benefit most by CoreNEURON. The Ring network model has computations only from passive dendrites and active soma.

Intel KNL has 512-bit SIMD vectors and high bandwidth memory (MCDRAM). One needs to efficiently utilize these hardware features to achieve best performance. In the case of CoreNEURON, NMODL generated code is auto-vectorized by the compiler and has *SoA* memory layout to provide uniform, contiguous memory access. NEURON uses *AoS* memory layout which results in strided memory accesses. Due to the lower clock frequency of KNL cores, the performance impact of non-vectorized code and strided memory accesses is high compared to other architectures. Hence CoreNEURON delivers better performance on KNL compared to NEURON. Note that the Cortex+Plasticity and Hippocampus models have relatively less improvement (2-4x) compared to the Cortex model (3-7x). This is because some of the channel and synapse descriptions explicitly request integration methods that present compilers cannot efficiently vectorize. Alternative code generation for these methods is being considered.

On the BlueGene/Q platform the speedup with most of the models is limited to 2x. This is because the IBM XL compiler is not able to vectorize most of the channel and synapse kernels. Observed performance improvement on this platform is due to more efficient memory accesses from the *SoA* layout discussed in the section 4.2.

GPU support has been recently added to CoreNEURON. Two models used in this benchmark, Cortex+Plasticity and Hippocampus, use legacy HOC based stimulus implementations which are not adapted for GPU yet. The Ring network model has large number of identical cells which suits SIMD computations on GPU and hence shows significant performance improvement compared to other architectures. The Traub model has a small number of cells exposing limited parallelism and the Dentate model has gap junctions which require copying of voltages between CPU and GPU every timestep. This limits the performance improvement on GPU.

The reduction in the memory footprint of models translates directly into benefits for users of large-scale models. For example, while models of the size of Cortex + Plasticity and Hippocampus models had a memory requirement when using NEURON that necessitated a minimum of 2,048 nodes on an IBM BlueGene/Q system, can now run on the same system requiring only 128 or 256 nodes for the Cortex+Plasticity and Hippocampus model respectively when using the *CoreNEURON Offline Mode*. This is a significant usability improvement and translates directly into a better use of a user's compute allocation.

**Figure 8:**
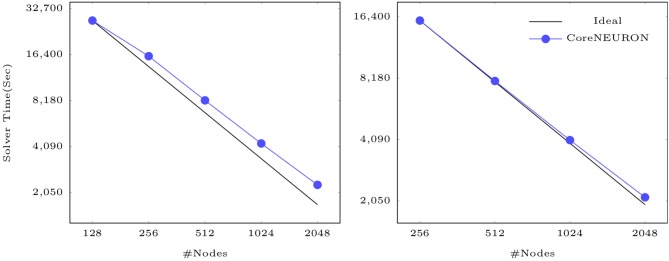
Strong scaling of CoreNEURON on the BB4 system for two large scale models listed in Table 1: the Cortex+Plasticity model with 219 k neurons (on the left) and the Hippocampus CA1 model with 789 k neurons (on the right).

Finally, Figure 8 shows that CoreNEURON maintains good strong scaling properties for large models, as illustrated on the example of the Cortex+Plasticity and Hippocampus models simulating one second of biological time on an IBM BlueGene/Q system. As these models are compute intensive and a small fraction of execution time is spent in spike communication, the scaling behavior depends on how well a given number of cells can be distributed across the available number of ranks to yield good load balance. Both models show excellent strong scaling behavior up to 2,048 nodes. Due to the large size range of morpho-electrical neuron types, at least 7–10 cells per MPI process are required to achieve good load balance. With 32,000 MPI processes (16 ranks per node) and about 219,000 cells of Cortex+Plasticity, the load balance is not as good as with the Hippocampus model of about 789,000 cells. Hence, the Cortex+Plasticity model exhibits poorer scaling behavior compared to the Hippocampus model.

## 6. Discussion

Modern compute architectures can significantly boost application performance and the study of the brain *in silico* is in dire need to embrace this capability and trend. Accordingly, the widely used NEURON simulator that supports a large variety of models has been over the years successfully adapted to embrace massively parallel architectures, but its primary design goals were to allow for a flexible definition of models and interactive introspection thereof. It was neither designed for ultimate memory efficiency nor maximal performance. However, the larger and more detailed the models get, the larger are the resource requirements to simulate those models. Eventually, the costs of a system required for an un-optimized simulator should be weighed against the effort of reworking the simulator to make more efficient use of resources. In the context of the Blue Brain Project we took the decision to contribute to making the NEURON simulator more efficient for large models, effectively leading to reduced resource requirements, faster time-to-solution, or simply the capability to run bigger models on a given resource.

### 6.1. Compatibility With Existing NEURON Models

As the neuroscience community has developed and shared thousands of models with NEURON, compatibility and reproducibility has been one of the primary design goals. To maintain maximal compatibility, we chose the path of extracting the computational relevant parts of NEURON into a library called CoreNEURON and adapting it to exploit the computational features of modern compute architectures. This is a different path as for example taken by the Arbor (Akar et al., 2019) which started its developments from scratch. While such a fresh start has its benefits in terms of designing for future architectures from the start, we can show that the transformation approach we took immediately gives compatibility with a large number of existing NEURON models with minimal modification. Currently, CoreNEURON does not handle non thread-safe models and requires NMODL modifications if constructs like POINTER are used. We are working on handling such models transparently.

### 6.2. Flexibility for Model Building and Efficiency for Model Simulation

Many modeling workflows related to detailed brain models require flexibility for quickly inspecting and changing the models. By extracting the compute engine from the NEURON simulator environment and providing different methods of how it can interact with the NEURON simulator, one maintains the flexibility of NEURON for the construction of the models and can more easily apply optimizations to the compute engine for the costly simulation phase. The *Offline* execution mode of CoreNEURON provides flexibility to build and simulate large network models that cannot be simulated with NEURON. Thanks to the use of MPI, and the OpenMP and OpenACC programming models to achieve portability across different architectures such as multi-core, many-core CPUs, and GPUs.

### 6.3. Reduced Memory and Faster Time-to-Solution

The data structure changes allow CoreNEURON to use significantly less memory compared to NEURON. The *SoA* memory layout and code vectorization allow CoreNEURON to simulate modelsí efficiently. We benchmarked five different network models on different architectures showing 4-7x memory usage reduction and 2-7x execution time improvement.

### 6.4. Future Work

We discussed the implementation of the most significant changes and optimizations in NEURON and CoreNEURON. Although CoreNEURON can be used transparently within NEURON, users cannot currently access or modify model properties during integration. Work is ongoing in regard to bidirectional data copy routines activated by normal NEURON variable name evaluation and assignment syntax ranging in granularity from the entire model, to specific named arrays, down to individual variables. On the numerical side, CoreNEURON today supports network simulations using the fixed time step method but not the variable time step integration method (CVODE) (Cohen and Hindmarsh, 1996). The latter is rarely used in network simulations because state or parameter discontinuities in response to synaptic events demand continuous re-initialization of variable step integrators. Research is ongoing on how to improve the applicability of variable time step schemes in network simulation and can be considered for inclusion at a later stage. Currently, mapping of multiple MPI ranks to GPUs is not optimal and this will be addressed in future releases. Lastly, the NMODL source-to-source translator will be improved to generate efficient code for stiff, coupled, non-linear gating state complexes that require the *derivimplicit* integration method as well as the generation of optimal code for GPUs.

### 6.5. Availability

CoreNEURON and code generation program MOD2C are open sourced and available on GitHub (CoreNEURON GitHub Repository, 2019; MOD2C GitHub Repository, 2019).

## Data Availability Statement

The datasets relevant for the main conclusions of this study (i.e,. Ring model, Traub model, and Dentate gyrus model) can be accessed on GitHub (Raikov and Hines, 2016; Hines, 2017a,b). The remaining supplementary datasets that further support the generalization were granted by the respective research groups prior to their publication.

## Author Contributions

MH was creator of NEURON simulator and developing CoreNEURON library with PK. JF and AO contributed to the development of core features in CoreNEURON. JK has integrated CoreNEURON with NEURON using HOC and Python scripting interface and helped in the validation of simulations. FD and FS has guided overall development and scientific roadmap of CoreNEURON. The final version of the article is written and edited jointly by all authors.

### Conflict of Interest

The authors declare that the research was conducted in the absence of any commercial or financial relationships that could be construed as a potential conflict of interest.

## References

[B1] Ábrahám E. Bekas C. Brandic I. Genaim S. Johnsen E. B. Kondov I. (2015). Preparing hpc applications for exascale: challenges and recommendations, in 2015 18th International Conference on Network-Based Information Systems (Taipei), 401–406.

[B2] Akar N. A. Cumming B. Karakasis V. Küsters A. Klijn W. Peyser A. (2019). Arbor — a morphologically-detailed neural network simulation library for contemporary high-performance computing architectures, in 2019 27th Euromicro International Conference on Parallel, Distributed and Network-Based Processing (PDP) (Pavia), 274–282.

[B3] Anastassiou C. A. Perin R. BuzsÃiki G. Markram H. Koch C. (2015). Cell type- and activity-dependent extracellular correlates of intracellular spiking. J. Neurophysiol. 114, 608–623. 10.1152/jn.00628.2014 25995352 PMC4509390

[B4] Arkhipov A. Gouwens N. W. Billeh Y. N. Gratiy S. Iyer R. Wei Z.. (2018). Visual physiology of the layer 4 cortical circuit *in silico*. PLOS Comput. Biol. 14, 1–47. 10.1371/journal.pcbi.1006535 30419013 PMC6258373

[B5] Blundell I. Brette R. Cleland T. A. Close T. G. Coca D. Davison A. P.. (2018). Code generation in computational neuroscience: a review of tools and techniques. Front. Neuroinform. 12:68. 10.3389/fninf.2018.00068 30455637 PMC6230720

[B6] Cohen S. D. Hindmarsh A. C. (1996). Cvode, a stiff/nonstiff ode solver in c. Comput. Phys. 10, 138–143.

[B7] CoreNEURON GitHub Repository (2019). CoreNEURON - Simulator Optimized for Large Scale Neural Network Simulations. Available online at: https://github.com/BlueBrain/CoreNeuron (accessed May 1, 2019).

[B8] Dagum L. Menon R. (1998). OpenMP: an industry standard API for shared-memory programming. IEEE Comput. Sci. Eng. 5, 46–55.

[B9] Dahmen D. van Albada S. J. Tetzlaff T. Hagen E. Grün S. Diesmann M.. (2016). Hybrid scheme for modeling local field potentials from point-neuron networks. Cereb. Cortex 26, 4461–4496. 10.1093/cercor/bhw237 27797828 PMC6193674

[B10] Davies S. N. (1992). Neural networks of the hippocampus. by Roger D. Traub and Richard Miles. pp. 281. Cambridge university press, 1991. isbn 0 521 36481 7. Exp. Physiol. 77, 238–238.

[B11] De Schutter E. Bower J. (1994). An active membrane model of the cerebellar purkinje cell i. simulation of current clamps in slice. J. Neurophysiol. 71, 375–400. 7512629 10.1152/jn.1994.71.1.375

[B12] Deco G. Jirsa V. K. Robinson P. A. Breakspear M. Friston K. (2008). The dynamic brain: from spiking neurons to neural masses and cortical fields. PLOS Comput. Biol. 4, 1–35. 10.1371/journal.pcbi.1000092 18769680 PMC2519166

[B13] Dyhrfjeld-Johnsen J. Santhakumar V. Morgan R. J. Huerta R. Tsimring L. Soltesz I. (2007). Topological determinants of epileptogenesis in large-scale structural and functional models of the dentate gyrus derived from experimental data. J. Neurophysiol. 97, 1566–1587. 10.1152/jn.00950.2006 17093119

[B14] Gal E. London M. Globerson A. Ramaswamy S. Reimann M. Muller E.. (2017). Rich cell-type-specific network topology in neocortical microcircuitry. Nat. Neurosci. 20, 1004–13. 10.1038/nn.4576 28581480

[B15] Gewaltig M.-O. Diesmann M. (2007). NEST (NEural simulation tool). Scholarpedia 2:1430 10.4249/scholarpedia.1430

[B16] Haring R. Ohmacht M. Fox T. Gschwind M. Satterfield D. Sugavanam K. (2012). The IBM blue gene/Q compute chip. IEEE Micro 32, 48–60. 10.1109/MM.2011.108

[B17] Hepburn I. Chen W. Schutter E. D. (2016). Accurate reaction-diffusion operator splitting on tetrahedral meshes for parallel stochastic molecular simulations. J. Chem. Phys. 145:054118. 27497550 10.1063/1.4960034

[B18] Hewlett Packard Enterprise (2019). HPE SGI 8600 System. Available online at: https://h20195.www2.hpe.com/V2/getpdf.aspx/A00016640ENW.pdf (accessed May 1, 2019).

[B19] Hines M. (1993). NEURON—a program for simulation of nerve equations, in Neural Systems: Analysis and Modeling, ed Eeckman F. (Norwell, MA: Kluwer), 127–136.

[B20] Hines M. (2014). NEURON GPU Implementation. Available online at: https://bitbucket.org/nrnhines/nrngpu (accessed May 1, 2019).

[B21] Hines M. (2017a). Ring Network Model of Ball-and-Stick neurons. Available online at: https://github.com/nrnhines/ringtest (accessed May 1, 2019).

[B22] Hines M. (2017b). Traub 2005 model for CoreNEURON. Available online at: https://github.com/nrnhines/nrntraub (accessed May 1, 2019).

[B23] Hines M. (2019). NMODL User Guide. Available online at: https://www.neuron.yale.edu/neuron/static/py_doc/modelspec/programmatic/mechanisms/nmodl.html (accessed May 1, 2019).

[B24] Hines M. Kumar S. Schurmann F. (2011). Comparison of neuronal spike exchange methods on a blue gene/p supercomputer. Front. Comput. Neurosci. 5:49. 10.3389/fncom.2011.00049 22121345 PMC3219917

[B25] Hines M. L. Carnevale N. T. (1997). The neuron simulation environment. Neural Comput. 9, 1179–1209. 9248061 10.1162/neco.1997.9.6.1179

[B26] Hines M. L. Carnevale N. T. (2000). Expanding neuron's repertoire of mechanisms with nmodl. Neural Comput. 12, 995–1007. 10.1162/089976600300015475 10905805

[B27] Hines M. L. Markram H. Schürmann F. (2008). Fully implicit parallel simulation of single neurons. J. Comput. Neurosci. 25, 439–448. 10.1007/s10827-008-0087-5 18379867 PMC2760991

[B28] Human Brain project (2018). Community Models of Hippocampus. Available online at: https://www.humanbrainproject.eu/en/brain-simulation/hippocampus/ (accessed January 11, 2018).

[B29] Ippen T. Eppler J. M. Plesser H. E. Diesmann M. (2017). Constructing neuronal network models in massively parallel environments. Front. Neuroinform. 11:30. 10.3389/fninf.2017.00030 28559808 PMC5432669

[B30] Izhikevich E. M. Edelman G. M. (2008). Large-scale model of mammalian thalamocortical systems. Proc. Natl. Acad. Sci. U.S.A 105, 3593–3598. 10.1073/pnas.0712231105 18292226 PMC2265160

[B31] Jolivet R. Coggan J. S. Allaman I. Magistretti P. J. (2015). Multi-timescale modeling of activity-dependent metabolic coupling in the neuron-glia-vasculature ensemble. PLOS Comput. Biol. 11, 1–23. 10.1371/journal.pcbi.1004036 25719367 PMC4342167

[B32] Kumar S. Heidelberger P. Chen D. Hines M. (2010). Optimization of applications with non-blocking neighborhood collectives via multisends on the blue gene/p supercomputer. IPDPS 2010, 1–11. 10.1109/IPDPS.2010.5470407 21666880 PMC3111918

[B33] Kumbhar P. Hines M. Fouriaux J. Ovcharenko A. King J. Delalondre F. (2019). CoreNEURON : an optimized compute engine for the NEURON Simulator. arXiv:1901.10975. 10.3389/fninf.2019.00063 PMC6763692 31616273

[B34] Kumbhar P. Hines M. Ovcharenko A. Mallon D. A. King J. Sainz F. (2016). Leveraging a Cluster-Booster Architecture for Brain-Scale Simulations (Frankfurt: Springer International Publishing), 363–380.

[B35] Lange M. Gorman G. Weiland M. Mitchell L. Southern J. (2013). Achieving efficient strong scaling with petsc using hybrid mpi/openmp optimisation. in Supercomputing eds Kunkel J. M. Ludwig T. Meuer H. W. (Berlin; Heidelberg: Springer), 97–108.

[B36] Levine J. John L. (2009). Flex & Bison, 1st Edn. O'Reilly Media, Inc. Available online at: http://shop.oreilly.com/product/9780596155988.do

[B37] Lindroos R. Dorst M. C. Du K. FilipoviÄ M. Keller D. Ketzef M.. (2018). Basal ganglia neuromodulation over multiple temporal and structural scales simulations of direct pathway msns investigate the fast onset of dopaminergic effects and predict the role of kv4.2. Front. Neural Circ. 12:3. 10.3389/fncir.2018.00003 29467627 PMC5808142

[B38] Mainen Z. F. Sejnowski T. J. (1996). Influence of dendritic structure on firing pattern in model neocortical neurons. Nature 382, 363–366. 8684467 10.1038/382363a0

[B39] Markram H. Muller E. Ramaswamy S. Reimann M. W. Abdellah M. Sanchez C. A.. (2015). Reconstruction and simulation of neocortical microcircuitry. Cell 163, 456–492. 10.1016/j.cell.2015.09.029 26451489

[B40] Migliore M. Cannia C. Lytton W. W. Markram H. Hines M. L. (2006). Parallel network simulations with nEURON. J. Comput. Neurosci. 21, 119–129. 10.1007/s10827-006-7949-5 16732488 PMC2655137

[B41] MOD2C GitHub Repository (2019). MOD2C - Converter for Mod Files to C Code. Available online at: http://github.com/BlueBrain/mod2c (accessed May 1, 2019).

[B42] Nichols B. Buttlar D. Farrell J. (1996). Pthreads Programming: A POSIX Standard for Better Multiprocessing. O'Reilly Media, Inc. Available online at: http://shop.oreilly.com/product/9781565921153.do

[B43] NVIDIA Corporation (2006–2017). CUDA. Available online at: https://developer.nvidia.com/about-cuda (accessed January 24, 2019).

[B44] NVIDIA Corporation (2012). TESLA K20X GPU Accelerator. Available online at: https://www.nvidia.com/content/pdf/kepler/tesla-k20x-bd-06397-001-v07.pdf (accessed April 4, 2019).

[B45] ParallelContext (2019). NEURON User Guide. Available online at: https://www.neuron.yale.edu/neuron/static/docs/help/neuron/neuron/classes/parcon.html#psolve (accessed May 1, 2019).

[B46] POINTER (2019). NMODL User Guide. Available online at: https://www.neuron.yale.edu/neuron/static/py_doc/modelspec/programmatic/mechanisms/nmodl.html#pointer (accessed May 1, 2019).

[B47] Potjans T. C. Diesmann M. (2012). The cell-type specific cortical microcircuit: Relating structure and activity in a full-scale spiking network model. Cereb. Cortex 24, 785–806. 10.1093/cercor/bhs358 23203991 PMC3920768

[B48] Raikov I. Hines M. (2016). Model of a Dentate Granule Cells Adapted for CoreNEURON. Available online at: https://github.com/pramodk/reduced_dentate (accessed May 1, 2019).

[B49] Reimann M. W. Nolte M. Scolamiero M. Turner K. Perin R. Chindemi G.. (2017). Cliques of neurons bound into cavities provide a missing link between structure and function. Front. Comput. Neurosci. 11:48. 10.3389/fncom.2017.00048 28659782 PMC5467434

[B50] Salmon J. K. Moraes M. A. Dror R. O. Shaw D. E. (2011). Parallel random numbers: as easy as 1, 2, 3, in Proceedings of 2011 International Conference for High Performance Computing, Networking, Storage and Analysis, SC '11 (New York, NY:ACM), 16:1–16:12.

[B51] Sanz-Leon P. Knock S. A. Spiegler A. Jirsa V. K. (2015). Mathematical framework for large-scale brain network modeling in the virtual brain. NeuroImage 111, 385–430. 10.1016/j.neuroimage.2015.01.002 25592995

[B52] Schulz M. Bronevetsky G. Fernandes R. Marques D. Pingali K. Stodghill P. (2004). Implementation and evaluation of a scalable application-level checkpoint-recovery scheme for MPI programs, in SC '04: Proceedings of the 2004 ACM/IEEE Conference on Supercomputing (Pittsburgh, PA: IEEE), 38–38. 10.1109/SC.2004.29

[B53] Soltesz Lab (2019). Available online at: http://med.stanford.edu/ivansolteszlab/front-page.html (accessed May 1, 2019).

[B54] THREADSAFE (2019). NEURON User Guide. Available online at: https://www.neuron.yale.edu/neuron/static/py_doc/modelspec/programmatic/network/parcon.html#ParallelContext.Threads (accessed May 1, 2019).

[B55] Traub R. D. Contreras D. Cunningham M. O. Murray H. LeBeau F. E. N. Roopun A.. (2005). Single-column thalamocortical network model exhibiting gamma oscillations, sleep spindles, and epileptogenic bursts. J. Neurophysiol. 93, 2194–2232. 10.1152/jn.00983.2004 15525801

[B56] Tuckwell H. C. (2005). Introduction to Theoretical Neurobiology: Volume 2, Nonlinear and Stochastic Theories, Volume 8. Cambridge University Press. Available online at: https://www.cambridge.org/fr/academic/subjects/mathematics/mathematical-biology/introduction-theoretical-neurobiology-volume-2?format=PB&isbn=9780521019323

[B57] Valero-Lara P. Martínez-Perez I. Peña A. J. Martorell X. Sirvent R. Labarta J. (2017). cuHinesBatch: Solving multiple hines systems on GPUs human brain project. Proc. Comput. Sci. 108, 566–575. 10.1016/j.procs.2017.05.145

[B58] Wikipedia (2006). NVIDIA CUDA. Available online at: https://en.wikipedia.org/wiki/CUDA (accessed April 20, 2019).

[B59] Wikipedia (2012). OpenACC. Available online at: https://en.wikipedia.org/wiki/OpenACC (accessed April 20, 2019).

[B60] Wils S. De Schutter E. (2009). Steps: modeling and simulating complex reaction-diffusion systems with python. Front. Neuroinform. 3:15. 10.3389/neuro.11.015.2009 19623245 PMC2706651

