# Supplementary material for: CoreNEURON : An Optimized Compute Engine for the NEURON Simulator
Source: Front Neuroinform. 2019 Sep 19;13:63. doi: 10.3389/fninf.2019.00063 (PMC6763692; doi:10.3389/fninf.2019.00063)
Supplement: Supplementary file 1 [file Table_1.pdf]

Supplementary Material

0.1 Supplementary Tables

Table S1. Different semantics types introduced to facilitate memory *serialization* and *deserialization* are listed with their purpose. For example, *area* and *pntproc* are to identify compartment area (*double* value) and point process (*Point_process* object) respectively.

Semantic Name	Purpose
area	area of the compartment or segment
iontype	type of ion used (calcium, sodium, potassium)
cvodeieq	element on event queue
netsend	network send event for communication
pointer/corepointer	pointer variable used in NEURON/CoreNEURON
pntproc	point process object
watch	element used as WATCH statement
diam	diameter of the compartment or segment
